# Predictive factors of left atrial spontaneous echo contrast in patients with rheumatic mitral valve stenosis: a retrospective study of 159 patients

**DOI:** 10.1186/1755-7682-7-32

**Published:** 2014-06-25

**Authors:** Sanaa Drissi, Hicham Sabor, Ahlam Ounsy, Najat Mouine, Mohamed Sabry, Aatif Benyass, El Mehdi Zbir, Konate Lassana, Naima Elhaithem

**Affiliations:** 1 Department of Cardiology, Mohammed V Military Teaching Hospital, Rabat, Morocco; 2 Department of Cardiology, Avicenne University Hospital, Rabat, Morocco

**Keywords:** Mitral stenosis, Left atrial spontaneous contrast, Transesophageal echocardiography, Anticoagulation

## Abstract

**Background:**

Mitral valve stenosis is a common manifestation of chronic rheumatic heart disease. The presence of spontaneous echo contrast in the left atrium and left atrial appendage has been reported to be an independent predictor of thrombo-embolic risk in patients with mitral stenosis. The objective of this study was to retrospectively investigate various clinical and echocardiographic variables to predict the spontaneous echo contrast in these patients.

**Methodology:**

This is a bicentric retrospective study which includes 159 cases of symptomatic mitral stenosis from January 2011 to June 2012. All of the patients had transthoracic and transesophageal echocardiography. Patients who had significant mitral regurgitation (> Grade I), significant aortic valve disease, previous mitral valvulotomy and anticoagulation or antiplatelet therapy were excluded from the study. Our study population was divided into two groups based on the presence (Group I) or absence (Group II) of spontaneous echo contrast.

**Result:**

Left atrial spontaneous contrast was present in 34.6% of cases. Patients in this group have more frequent atrial fibrillation (P = 0.001), larger left atrial area (P = 0.027) and diameter (P=0.023), smaller mitral valve area (P = 0.025), and higher mean transmitral diastolic gradient (p = 0.003) as compared to patients without spontaneous echo contrast. There were no significant differences in the mean age (p = 0.38), duration of symptoms (p = 0.4) and left ventricular ejection fraction (p = 0.7) between patients with and without spontaneous echo contrast. On multivariate analysis, only mitral valve area and transmitral diastolic gradient (OR: 18.753, 1.21, CI [1,838-191,332], [1,064-1,376], p: 0.013, 0.004, respectively) were found to be independently associated to the presence of spontaneous echo contrast.

**Conclusion:**

Patients with severe rheumatic mitral stenosis in atrial fibrillation or sinus rhythm have a higher risk of developing spontaneous echo contrast. These patients might benefit from prophylactic anticoagulation. The long-term outcomes can be ascertained in a study over a longer period and with periodic follow-up.

## Introduction

Rheumatic fever primarily affects children in developing countries, especially where poverty is widespread. Globally, almost 2% of deaths from cardiovascular diseases are related to rheumatic heart disease, while 42% of deaths from cardiovascular diseases is related to ischaemic heart disease, and 34% to cerebrovascular disease [1,2]. Rheumatic mitral valve stenosis is a common manifestation of chronic rheumatic heart disease.

Spontaneous echo contrast (SEC) is an echogenic swirling pattern of blood flow caused by an increased ultrasonic back-scatter from aggregation of the cellular components of blood in conditions of blood stasis or low-velocity blood flow [3,4]. The incidence of spontaneous echo contrast in rheumatic mitral stenosis varies from 21% to 67% [5-8]. Patients with spontaneous echo contrast in the left atrium and left atrial appendage had a higher incidence of systemic embolization [5,9,10], which is major cause of morbidity and mortality [11-13].

Various factors determine individual risk for the development of spontaneous echo contrast in patients with rheumatic mitral stenosis, including mitral valve area (MVA) [7,11,14,15], mitral valve gradient [14], left atrial size and area [7,16-18], atrial fibrillation [18,19], as well as some hematologic factors such as hematocrit and fibrinogen levels [20], duration of symptoms [21], older age [10-21] and severity of mitral stenosis [16-22].

Our study analyzes clinical and echocardiographic factors which have previously been implicated in the development of left atrial spontaneous echo contrast in a large population of patients with symptomatic rheumatic mitral stenosis. All of the patients were retrospectively examined by transthoracic and transesophageal echocardiograph.

## Material and methods

This bicentric retrospective study included 159 patients who had been admitted for symptomatic mitral stenosis in Avicenne University Hospital and Mohamed V Military Teaching Hospital in Rabat, Morocco, from January 2011 to June 2012. All of the patients had a clinical examination, EKG, transthoracic echocardiography, and transesophageal echocardiography. Patients who had significant mitral regurgitation (> Grade I), significant aortic valve disease, previous closed mitral valvulotomy and anticoagulation or antiplatelet therapy were excluded from the study.

Transthoracic echocardiography measurements were LA dimension, LA area and mitral valve (MV) areas (by planimetry and by pressure half time method). All measurements were taken according to the recommendations of the American Society of Echocardiography [16]. Transesophageal echocardiography was performed under local anesthesia. Patients were divided into two groups based on the presence (Group I) or absence (Group II) of SEC in the left appendage and left atrium. All patients were followed up for a period of one to six months after treatment by mitral valvulotomy or prosthesis.

Statistical Package for Social Sciences 10 was used to perform the statistical analysis. Quantitative variables were expressed as mean ± standard deviation. For comparison of measurements between Groups I and II, Student’s *t*-test was used for continuous variables and *χ*^2^-test for categorical variables, and values were considered significant when the *P* value was <0.05. Univariate, multiple logistic regression analysis was done for all variables to determine the factors that independently predict the presence of spontaneous contrast.

## Results

159 subjects (26 male and 133 female) were assessed over a 17-month period. All of them had severe rheumatic mitral stenosis with no significant mitral regurgitation. The mean age was 40.87 ± 12.19 years (range 11–75 years). A history of rheumatic fever was reported in 35.2% of patients. The disease was revealed by dyspnea and ischemic stroke in 87% and 4.4% of patients, respectively.

Fifty-five patients had demonstrable spontaneous echo contrast (Group I) in the left atrium and left atrial appendage in 31 and 24 cases, respectively. Six cases of thrombus were detected in Group I. Table 1 compares clinical details and echographic measurements in the two groups of patients. There was no significant difference in the mean age (p = 0.389), gender (p = 0.37), and duration of symptoms (p = 0.4) between the two groups.

**Table 1:** Comparison between patients with (Group I) and without (Group II) spontaneous echo contrast

	**Group I**	**Group II**	***P-*** **value**
	n *= 55*	n *= 104*	
**Age (years)**	42.02 ± 10.57	40.26 ± 12.97	0.389
**Gender**	7 male	19 male	0.37
	48 female	85 female	
**Duration of symptoms (months)**	57.56 ± 9.71	56.27 ± 9.20	0.4
**AF**^**1**^	33 (60%)	33 (31.7%)	0.001
**MVA**^**2 **^**(cm**^**2**^**)**	0.91 ± 0.23	1 ± 0.22 cm^2^	0.025
**MDG**^**3 **^**(mmHg)**	13.91 ± 5.43	11.77 ± 3.61	0.003
**LVEF**^**4 **^**(%)**	65.22 ± 5.63	64.88 ± 5.49	0.7
**LAD**^**5 **^**(mm)**	51.40 ± 7.74	48.39 ± 7.9	0.023
**LA**^**6 **^**(cm**^**2**^**)**	33.09 ± 6.39	30.83 ± 7.08	0.027

^1^Atrial fibrillation; ^2^Mitral valve area; ^3^Mitral diastolic gradient; ^4^Left ventricular ejection fraction; ^5^Left atrial diameter; ^6^Left atrial area.

Compared to patients without SEC, patients with SEC have more frequent atrial fibrillation (P = 0.001), smaller mitral valve area (p = 0.025), high transmitral diastolic gradient (p = 0,003), larger left atrium area (p = 0.023), and diameter (p = 0.027). The difference in left ventricular ejection fraction between two groups was not statistically significant (p = 0.7).

Univariate analysis regression is presented in Table 2. Results given show that atrial fibrillation (p = 0.001), lower mitral valve area (p = 0.027), higher transmitral diastolic gradient (p = 0.001) and left atrial diameter (p = 0.027) were significantly correlated to the presence of spontaneous echo contrast. Left atrial area was also higher in patients with spontaneous echo contrast but was not statistically significant (p = 0, 27).

**Table 2:** Correlate parameters to spontaneous echo contrast on univariate analysis between two groups

**Variables**	**OR**	**IC**	**P-value**
**Age**	1.02	[0.98–1.03]	0.38
**Sex**	0.65	[0.25–1.66]	0.37
**Duration of symptoms (months)**	1.014	[0.978–1.05]	0.4
**AF**	3.22	[1.63–6.366]	0.001
**LA**	1.027	[0.97–1.077]	0.272
**LAD**	1.04	[1.005–1.095]	0.027
**MVA**	1.89	[0.043–0.830]	0.027
**MDG**	0.9	[0.834–0.977]	0.001
**LVEF**	1.011	[0.953–1.073]	0.709

However, as shown in Table 3, multivariate regression analysis revealed that only mitral valve area (p *=* 0.013) and transmitral diastolic gradient (p = 0.004) were found to be independently associated with the presence of spontaneous echo contrast in the left atrial and left atrial.

**Table 3:** Correlate parameters on multivariate analysis regression between two groups

**Variables**	**OR**	**CI**	**P**
**AF**	0.6	[0.231–1.557]	0.294
**LAD**	0.938	[0.858–1.025]	0.15
**MVA**	18.753	[1.838–191.332]	0,013
**MDG**	1.210	[1.064–1.376]	0.004

## Discussion

In this retrospective study of patients with rheumatic severe mitral stenosis, we evaluated several clinical and echocardiographic variables which correlated with the presence of left atrial and left atrial appendage spontaneous echo contrast.

The presence of spontaneous echo contrast has been reported to be an independent predictor of thrombo-embolic risk in patients with rheumatic mitral valve disease [16,23,24]. In a systematic review of literature on factors predictive of the development of spontaneous echo contrast in patients with rheumatic mitral stenosis, several variables have been reported. Variables include mitral valve area (MVA) [7,11,14,15] and gradient [12], left atrial size and area [7,16-18], atrial fibrillation [18,19], hematologic factors such as hematocrit and fibrinogen levels [20], duration of symptoms [21], older age [10-21], and severity of mitral stenosis [16-22].

Our patient population was younger with a female predominance, but there were no significant differences in age and gender between two groups. This could be explained by the method of recruitment, which was done at Mohammed V Military Teaching Hospital and whose patient population is primarily composed of young male soldiers and military wives. Studies by Deveral [13] and Fatkin [24] produced results similar to ours. In their studies, there was no statistical significant difference in the age between groups with and without spontaneous echo contrast.

Kewal et al. [18] demonstrated that spontaneous echo contrast was present in older patients (31 ± 10.4 vs. 27.8 ± 8.3 years, *P* < 0.01), although their patient population was also younger. This was due to a referral bias compounded by the aggressive nature of disease in the Indian subcontinent and echocardiography performed later.

The duration of symptoms did not correlate with the presence of left atrial and left atrial appendage spontaneous echo contrast on multivariate analysis in our study. This may mean that stasis of blood in the left atrium occurs relatively early in patients with mitral stenosis. Stasis of blood has been widely thought to be responsible for the appearance of left atrial spontaneous echo contrast. A review of literature supports our results [7,16-23].

Earlier studies [5-8] have observed that atrial fibrillation was associated with left atrial spontaneous echo contrast. Similarly, in a recent prospective study, Kewal et al. [18] illustrated that the presence of left atrial spontaneous echo contrast was much more common in patients with atrial fibrillation (48.6 vs. 9.7%, *P* < 0.0001). On multiple regression and discriminant function analysis, atrial fibrillation alone was found to be an independent clinical predictor of left atrial spontaneous echo contrast.

We found that atrial fibrillation was positively correlated with the presence of spontaneous echo contrast. However, when all meaningful variables were included in multivariate analysis, this parameter was not significant, suggesting that other echographic variables, such as mitral valve area and diastolic gradient, had a more influential affect.

In the present study, all patients underwent a routine transesophageal echocardiography following transthoracic echocardiography. We measured MV area using two methods, computed planimetry and pressure half time method.

We found that left atrial size, mitral valve area and mean diastolic gradient were significantly correlated with the presence of left atrial spontaneous echo contrast. Yet, after multiple regression analysis, only mitral valve area and diastolic gradient (p: 0.013, 0.004) respectively, were found to be independently related to the presence of spontaneous echo contrast.

Several studies have shown that spontaneous echo contrast (SEC) is increasingly prevalent with decreased mitral valve (MV) area. Kasliwal et al. [25] reported mean MV area of 1.07 ± 0.33 cm^2^ in patients with SEC and 1.32 ± 0.45 cm^2^ in those without it. Black et al. [23] found that MV area of 1.1 ± 0.3 cm^2^ was predictive of SEC. Beppu et al. [7] classified their cases into three categories on basis of mean MV area and SEC: 0.7 cm^2^ with heavy SEC, 1.1 cm^2^ with mild SEC, and 1.5 cm^2^ with no SEC.

In our study, it’s possible that a mean mitral valve area of 0.91 ± 0.23 cm^2^ exposes the patient to a higher risk of developing spontaneous echo contrast. In contrast to previous studies conducted by Beppu et al. [7], we have not classified the severity of SEC since we feel that categorizing SEC may not aid in clinical decision-making.

The mitral diastolic gradient was the second independent variable found to be an independent predictor of SEC in our patients, with a mean of 13.91 ± 5.43 cm^2^.

This data could be attributed to the fact that all of our patients had severe mitral stenosis, without associated regurgitation, and were already with high risk for formation of spontaneous echo contrast.

An earlier study conducted by Bernstein et al. [14] supports our results. The authors evaluated the correlates of spontaneous echo contrast in mitral stenosis and normal sinus rhythm. Their results show that mean transmitral gradient was significantly higher in Group 1 with contrast (13.6 +/- 5.2 mm Hg) than in Group 2 without contrast (10.5 ± 4.9 mm Hg) (p < 0.05). Mitral valve area was also significantly smaller in Group 1 than in Group 2 (1.0 ± 0.5 vs. 1.4 ± 0.5 cm^2^; p < 0.02). They conclude that, in the left atrium in patients with mitral stenosis and normal sinus rhythm, spontaneous echo contrast is common and is associated with a significantly smaller mitral valve area and higher mitral gradient. Conversely, Kewal et al. [18] reported that there was no significant difference in the mean diastolic gradient between two groups with and without SEC (15.1 ± 4.8 vs. 15.2 ± 5.1 mmHg).

Finally, it must be said that left atrial and left atrial appendage spontaneous echo contrast was an important predictor of systemic embolization [16,23,24] in mitral stenosis, and should be an indication of the need to initiate anticoagulation.

## Conclusion

We concluded that in patients with rheumatic mitral stenosis, a small mitral area of 0.9 cm^2^ and a high transmitral diastolic gradient of 13 mmHg were independent predictive of spontaneous echo contrast in the left atrium and left atrial appendage. It’s possible that these patient although normal sinus rhythms have a higher risk to develop spontaneous echo contrast, and might benefit from prophylactic anticoagulation. The application of this data to clinical practice will require a larger sample size, longer study duration, and follow up.

### Limitation of our study

The study was retrospective, with short duration. The discriminate function analysis was not used. Measures of thromboembolic risk like left atrial appendage size, and filling and emptying velocities were not studied. Since the relation of spontaneous echo contrast with systemic embolic event was not the objective of our study. A larger prospective study with longer duration and follow up could probably address.

### Consent (Adult)

A written informed consent was obtained from all patients for the publication of this paper.

## Competing interests

The authors declare that they have no competing interest.

## Authors’ contributions

HS participated in the sequence alignment and drafted the manuscript. AO participated in the sequence alignment and drafted the manuscript. NM participated in the sequence alignment and drafted the manuscript. MS participated in the sequence alignment and drafted the manuscript. AB participated in the sequence alignment and drafted the manuscript. EZ participated in the sequence alignment and drafted the manuscript. LK participated in the sequence alignment and drafted the manuscript. participated in the sequence alignment and drafted the manuscript. NE participated in the sequence alignment and drafted the manuscript. All authors read and approved the final manuscript.
